# Proteomic Analysis Reveals Proteins Involved in Seed Imbibition under Salt Stress in Rice

**DOI:** 10.3389/fpls.2016.02006

**Published:** 2017-01-05

**Authors:** Enshun Xu, Mingming Chen, Hui He, Chengfang Zhan, Yanhao Cheng, Hongsheng Zhang, Zhoufei Wang

**Affiliations:** ^1^The Laboratory of Seed Science and Technology, State Key Laboratory of Crop Genetics and Germplasm Enhancement, Jiangsu Collaborative Innovation Center for Modern Crop Production, Nanjing Agricultural UniversityNanjing, China; ^2^Department of Plant Science, College of Biological Sciences, Henan Agricultural UniversityZhengzhou, China

**Keywords:** rice, seed germination, seed imbibition, comparative proteomics, salt stress

## Abstract

Enhancement of salinity tolerance during seed germination is very important for direct seeding in rice. In this study, the salt-tolerant *japonica* landrace Jiucaiqing was used to determine the regulators that are involved in seed imbibition under salt stress. Briefly, the comparative proteomic analysis was conducted between dry (0 h) and imbibed (24 h) seeds with 150 mM NaCl. Under salt stress, the uptake of water increased rapidly before 24 h imbibition (Phase I), followed by a plateau of seed imbibition from 24 to 96 h imbibition (Phase II). We identified 14 proteins involved in seed imbibition, in which the majority of these proteins were involved in energy supply and storage protein. The early imbibition process was mediated by protein catabolism; the most of proteins were down-regulated after 24 h imbibition. Eleven genes in salt stress treated seeds were expressed early during the seed imbibition in comparison to control seeds. By comparison, 2,3-bisphosphoglycerate-independent phosphoglycerate mutase (*BPM*), glutelin (*GLU2.2* and *GLU2.3*), glucose-1-phosphate adenylyltransferase large subunit (*GAS8*), and cupin domain containing protein (*CDP3.1* and *CDP3.2*) were near the regions of quantitative trait loci (QTLs) for seed dormancy, seed reserve utilization, and seed germination in Jiucaiqing. In particular, *CDP3.1* was co-located in the region of *qIR-3* for imbibition rate, and *qGP-3* for germination percentage. The role of *CDP3.1* was verified in enhancing seed germination under salt stress using T-DNA mutant. The identified proteins might be applicable for the improvement of seed germination under salt stress in rice.

## Introduction

Rice (*Oryza sativa* L.) is a salt-sensitive crop. However, about 30% of the rice growing area in the world were affected by salinity (Takehisa et al., 2004) due to defective irrigation and fertilization (Lin et al., 2004). Salinity causes water deficit, ion toxicity, and nutrient deficiency, which can result in growth and yield reduction and even plant death (Maggio et al., 2010). The direct seeding method recently has become increasingly important in many Asian countries due to its lower cost and operational simplicity (Fujino et al., 2004; Wang et al., 2011). Therefore, improving seed germination under salt stress is an important goal for rice breeding.

Understanding the mechanisms of seed germination is helpful to develop the elite varieties with high seed vigor. In recent years, several proteomics analyses of seed germination had been applied in rice (Sano et al., 2012; Han et al., 2014a,b; Xu et al., 2016; Zhang et al., 2016). For example, it was reported by Sano et al. (2012) that *de novo* transcription was not required for rice seed germination. They also showed that some of the germination-specific proteins involved in energy production and maintenance of cell structure was synthesized from long-lived mRNAs. In addition, it has been showed that the proteins involved in carbohydrate metabolism and protein synthesis/catabolism were predominantly increased during rice seed germination (Han et al., 2014b). The carbonylated proteins were found to be mainly involved in maintaining the levels of ROS, abscisic acid, and seed reserves during rice seed germination (Zhang et al., 2016). Several important proteins were observed to be associated with rice seed germination involved in metabolism, energy production, protein synthesis, and destination, storage protein, cell growth and division, signal transduction, cell defense, and rescue (Xu et al., 2016). However, the proteomics analyses of seed imbibition under salt stress is scarcely reported in rice.

Based on the uptake of water, seed imbibition can be divided into three phases—a rapid uptake of water (Phase I), followed by a plateau phase of water uptake (Phase II), and a rapid uptake of water with the initiation of growth (Phase III; Wang et al., 2011; Cheng et al., 2017). During seed's imbibition, Phase II represents a critical development stage, in which all the necessary metabolic pathways and physiological processes are reactivated (Yang et al., 2007; He et al., 2011). It is found that germination process is highly disturbed by salt stress. The excessive concentrations of Na^+^ and Cl^−^ ions, reduction in osmotic potential, and ion toxic effects might be responsible for low seed germination under salt stress. Previously, we have identified one salt-tolerant *japonica* landrace Jiucaiqing from Taihu Lake valley in Jiangsu Province of China (Wang et al., 2011, 2012a,b). By using bi-parental population, we have identified several quantitative trait locus (QTLs) in Jiucaiqing for seed dormancy (Wang et al., 2014), seed reserve utilization (Cheng et al., 2013), seed vigor (Liu et al., 2014), and salt tolerance at seed germination (Wang et al., 2011) and seedling stages (Wang et al., 2012a,b). Recently, we found that the proteins associated with seed imbibition in distilled water could be categorized as carbohydrate and protein biosynthesis and metabolism-related, signaling-related, storage, and stress-related proteins in Jiucaiqing (Cheng et al., 2017). However, the regulators involved in seed imbibition under salt stress are still not certain.

In this study, two-dimensional gel electrophoresis (2-DE) was used to identify the proteins involved in seed imbibition under salt stress in rice. Fourteen proteins were identified. Among them, six proteins were near the regions of the previous QTLs for seed germination. Furthermore, one of the protein, cupin domain containing protein (*CDP3.1*), was chosen for verification and found to enhance seed germination under salt stress. These results will increase our understanding on rice seed germination under salt stress.

## Materials and methods

### Plant materials

The landrace Jiucaiqing (*Oryza sativa* spp. *japonica*) was used from the Taihu Lake valley in Jiangsu Province of China (Du et al., 2015; Cheng et al., 2017). Seeds were provided by the Laboratory of Seed Science and Technology in Nanjing Agricultural University (Nanjing, Jiangsu Province, China). All seeds were harvested at their maturity stage and dried at 42°C for 7 days (~13.5% moisture content) to break seed dormancy (Wang et al., 2011). The T-DNA insertion line PFG_1B-08438.L of *CDP3.1* (rice ssp. *japonica* cv. Dongjin) was ordered from the RISD database (Jeon et al., 2000; Jeong et al., 2002).

### Seed germination

Seed germination was conducted as previously described Cheng et al. (2017). Fifty seeds in replicate were imbibed in Petri dishes (*d* = 9 cm) under water or 150 mM NaCl conditions at 30 ± 1°C for 10 days. The solution was replaced every 24 h to maintain the NaCl concentration. The weight of imbibed seeds was recorded each 12 h to calculate the moisture content of seeds. Germination ability was observed every day to calculate germination percentage (GP) and seedling percentage (SP) (Du et al., 2015). Seeds were considered as germination when the radicle protruded (2 mm) through seed coat. Seedlings were considered to be established when the root length reached seed length and the shoot length reached half of seed length. Germination index (GI) was calculated by the method of (Wang et al., 2010): *GI* = Σ (*Gt*/*t*), where *Gt* is the number of the germinated seeds on day *t*. Three replicates were conducted.

### Protein extraction and quantification

Protein extraction and quantification were conducted as previously described by Cheng et al. (2017). Fifty seeds of each replicate were imbibed under 150 mM NaCl for 24 h, and then frozen in liquid nitrogen and stored at −80°C until further use along with the dry seeds as control. Total proteins were isolated using the tri-chloro-acetic acid (TCA)/acetone method according to He and Li (2008), and protein concentration was determined by the Bradford assay kit (Bio-Rad Laboratory, USA; Guo et al., 2004). Three biological replicates were conducted.

### Two-dimensional electrophoresis and protein identification

Two-dimensional electrophoresis and protein identification were conducted as previously described by Cheng et al. (2017). Total protein (100 μg) was loaded onto GE Healthcare 13 cm IPG gel strips (pH 3–10), which were rehydrated overnight. Isoelectric focusing (IEF) of the IPG strips was conducted by using IPGPhor II (GE Healthcare, USA) at 20°C for a total of 67.86 kVh. The second dimension was performed by using 15% SDS-PAGE gels on an Ettan Daltsix (GE Healthcare, USA). The running condition was 15 mA per gel for the first 30 min, followed by 30 mA per gel until the dye reached the bottom of gel. Gels were visualized with silver nitrate. The 2-DE gels were scanned with an Image Scanner and analyzed by using Image Master-Elite software (GE Healthcare, USA). The intensities of the differentially expressed protein spots on the 2-DE gels obtained in three independent experiments were quantitatively measured to obtain statistical information on variations in the protein levels. The spots with fold changes higher than 2.0 and the significant level at *P* < 0.01 by Student's *t*-test were considered to be differentially expressed proteins between the dry (0 h) and imbibed (24 h) seeds.

Those changed spots were manually excised from the gels and subjected to trypsinolysis according to standard techniques (Cheng et al., 2017). A total of 1 mL of peptides was loaded directly onto the MALDI target for analysis on the Ettan MALDI-TOF Pro system (GE Healthcare, USA) according to Fu et al. (2011). The online search engine Mascot (http://www.matrixscience.com) was used for the identification of proteins based on the NCBI non-redundant database (Fu et al., 2011). The searching parameters were as follows: all entries, parent ion mass tolerance at 50 ppm, MS/MS mass tolerance of 0.2 Da, cysteine carbamidomethylation as a fixed modification, and methionine oxidation as a variable modification. All the identified proteins should have at least one matched peptide with a probability more than 95%.

### Gene function annotation and gene meta-analysis

Gene ontology (GO) based enrichment tests were conducted using Singular Enrichment Analysis (SEA) in AgriGO toolkit (http://bioinfo.cau.edu.cn/agriGO/analysis.php) with a significant level FDR < 0.05 (Du et al., 2010). The gene expression at various developmental stages and tissues of rice was analyzed using the publicly available data from microarray platform–51K Affymetrix chips on Genevestigator (https://www.genevestigator.com/gv/plant.jsp; Cheng et al., 2017).

### Quantitative real-time PCR

Fifty seeds each replicate of Jiucaiqing were harvested after 0, 12, 24, and 48 h imbibition in distilled water and 150 mM NaCl solutions, and then were quick-frozen in liquid nitrogen and stored at −80°C for RNA extraction. All seeds were ground to a fine powder with a mortar and pestle on dry ice. Total RNA was extracted from approximately 80~100 mg powder using the RNeasy plant mini kit (Qiagen, www.qiagen.com) according to the manufacturer's protocol. First-strand cDNAs were synthesized from DNase I (Takara) treated total RNA using Thermo Scientific Revert Aid First Strand cDNA Synthesis kit following the manufacturer's instructions. The mRNA levels of genes were measured by quantitative real-time PCR (qRT-PCR) using CFX96 Real-time System (BIO-RAD, USA) with SYBR Green Mix (Vazyme). The rice 18S ribosomal RNA gene was used as an internal control. The PCR conditions were as follows: 95°C for 5 min, followed by 40 cycles of 95°C for 15 s and 60°C for 30 s. A final ramping stage of 65–95°C was performed to confirm the absence of multiple products and primer dimers. All of the primers used for qRT-PCR were designed according to http://quantprime.mpimp-golm.mpg.de/ (Supplementary Table 1). The relative quantification of the transcript levels was measured using the comparative Ct method (Livak and Schmittgen, 2001). Three biological replicates were conducted.

### Mutant identification

The T-DNA insertion site and homozygous line of *CDP3.1* was confirmed by PCR analysis of genomic DNA using gene-specific primers F1, 5′-AGGAATCCATTTCCGTTCTG- 3′, R1, 5′-TGGCAACAAAGTGAACAAGC-3′, and a T-DNA-specific primer T-DNA-F, 5′-ATGGCAGTGAATTAACATAGC- 3′. The PCR program was (1) 95°C for 5 min, (2) 95°C for 45 s, (3) 58°C for 45 s, (4) 72°C for 1 min, and (5) 72°C for 10 min. From steps 2–4, the cycles were repeated for 35 cycles. PCR products were directly sequenced and BLAST results showed that the T-DNA was inserted in the promotor of *CDP3.1* via NCBI BLAST of the rice genome database (https://blast.ncbi.nlm.nih.gov/Blast.cgi). Total RNAs were extracted from WT (wild type) and CDP3.1 mutant (*cdp3.1*) seeds for qRT-PCR analysis as described above. Three biological replicates were conducted.

### Evaluation of seedling growth and ion accumulation in tissues

One hundred seeds of wild type and *cdp3.1* in replicate were germinated under water and 150 mM NaCl conditions at 30 ± 1°C for 11 days. Ten seedlings in replicate were used to measure the fresh and dry weight of seedlings during 7–11 days germination stages. For Na^+^ accumulation analysis, the tissues of seedlings, including grains, shoots, and roots, were harvested and dried after 11 days germination. The seedlings were dried in an oven at 70°C for 12 h. Then, at least 0.5 g of dry tissues was used for acid digestion and ICP-AES analysis according to Møller et al. (2009). The Na^+^ accumulation in un-germinated dry seeds were also detected. The content of Na^+^ was expressed as mg per gram of dry mass. Each measurement was performed on three replicates.

### Data analysis

The experimental data were analyzed using the SPSS 19.0 software, and the differences between treatments were compared with Student's *t*-test at 5 and 1% levels of probability (Lai et al., 2016).

## Results

### Germination phenotype

**Figure 1:**
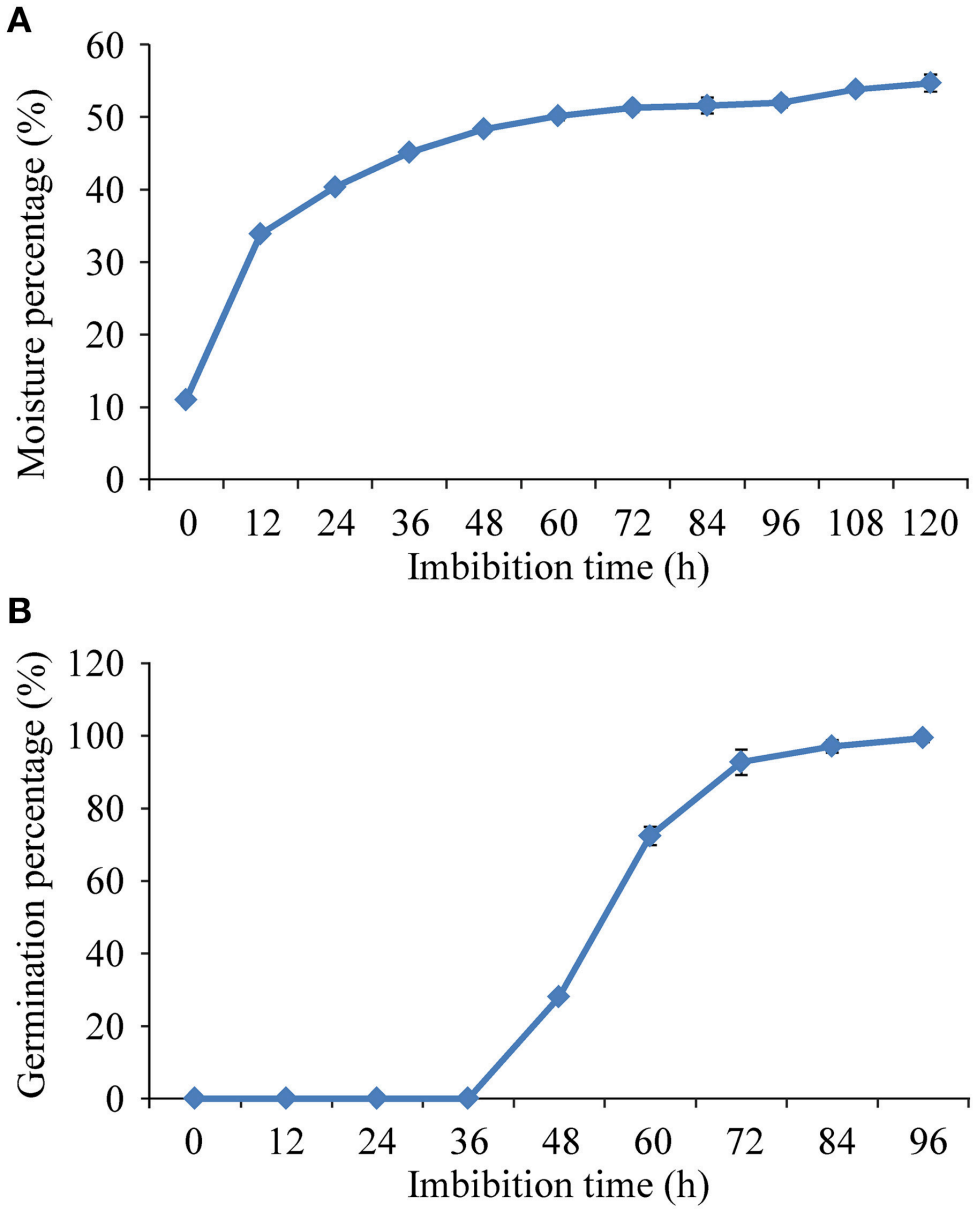
**Triphasic pattern of water uptake (A)** and changes of germination percentage **(B)** under 150 mM NaCl condition in rice.

Seeds depicted a triphasic pattern of water uptake during germination under salt stress (Figure 1A). The uptake of water increased rapidly before 24 h imbibition (Phase I), followed by a plateau of seed imbibition from 24 to 96 h imbibition (Phase II). During Phase II, the seeds radicles began to emerge after 36 h imbibition, and the maximum germination (99.3%) was attained after 96 h imbibition (Figure 1B). The imbibed seeds in the early Phase II (24 h) of imbibition were collected for further proteomic analysis.

### Seed imbibition responses proteins

A total of 60 proteins were identified with significant differences at a change ≥1.5-fold and *P* < 0.05 between dry and imbibed (24 h) seeds (Supplementary Figure 1). Among them, 18 protein with significant differences at a change ≥2.0-fold and *P* < 0.01 were chosen for further analysis, and 14 proteins were successfully identified (Table 1; Supplementary Figure 2; Supplementary Table 2). Most of proteins were decreased after 24 h imbibition, while only three proteins, spots 840, 1135 and 1462 (cupin domain containing protein), 1224 (60S acidic ribosomal protein), and 1981 (glutelin), were increased. Additionally, the same protein was identified in different spots, including glucose-1-phosphate adenylyltransferase large subunit (spots 488, 633, 706, and 669), cupin domain containing protein (spots 840, 1135, and 1462), granule-bound starch synthase I (spots 870, 981, and 1092), and glutelin (spots 1426, 1536, 1652, and 1981).

**Table 1:** **The successful identification of proteins differentially regulated in response to seed imbibition under salt stress in rice**.

**Spots^a^**	**Locus^b^**	**Protein MW^c^**	**Protein PI^d^**	**Protein Score**	**Protein Score C. I.%**	**Fold change^e^**	**Protein Name^f^**	**Gene Abbreviation^g^**
488	LOC_Os08g25734	68861.2	6.46	359	100	−2.38	Glucose-1-phosphate adenylyltransferase large subunit	*GAS8*
501	LOC_Os01g60190	60980	5.42	736	100	−2.36	2,3-bisphosphoglycerate-independent phosphoglycerate mutase	*BPM*
633	LOC_Os01g44220	57741.6	5.48	826	100	−5.11	Glucose-1-phosphate adenylyltransferase large subunit	*GAS1*
706	LOC_Os08g25734	53202.2	5.87	459	100	−2.95	Glucose-1-phosphate adenylyltransferase large subunit	*GAS8*
748	LOC_Os12g13320	52501.8	6.59	299	100	−2.82	Argininosuccinate synthase	*ARS*
840	LOC_Os03g57960	52436.3	6.78	552	100	2.93	Cupin domain containing protein	*CDP3.1*
870	LOC_Os06g04200	66978.1	8.34	83	99.9	−2.97	Granule-bound starch synthase I	*GSS*
959	LOC_Os02g07260	42195.5	5.64	195	100	−3.38	Phosphoglycerate kinase protein	*PKP*
981	LOC_Os06g04200	66978.1	8.34	78	99.6	−2.10	Granule-bound starch synthase I	*GSS*
1092	LOC_Os06g04200	66978.1	8.34	125	100	−2.66	Granule-bound starch synthase I	*GSS*
1135	LOC_Os03g57960	52436.3	6.78	447	100	2.70	Cupin domain containing protein	*CDP3.1*
1159	LOC_Os05g33570	88773.9	5.43	98	100	−2.00	Pyruvate phosphate dikinase	*PPD*
1224	LOC_Os11g04070	34470.1	5.38	198	100	3.44	60S acidic ribosomal protein	*ARP*
1426	LOC_Os02g14600	57153.2	9.56	246	100	−3.08	Glutelin	*GLU2.1*
1462	LOC_Os03g21790	61742.3	7.18	90	100	2.68	Cupin domain containing protein	*CDP3.2*
1536	LOC_Os03g31360	50691.4	8.74	99	100	−2.44	Glutelin	*GLU3*
1652	LOC_Os02g25640	55103.9	9.02	355	100	−3.48	Glutelin	*GLU2.2*
1981	LOC_Os02g16820	36037.8	6.6	80	99.7	9.93	Glutelin	*GLU2.3*

a *Numbers correspond to the 2-DE gels shown in Supplementary Figure 2*.

b *MSU gene symbol*.

c *Protein molecular weight (kDa)*.

d *Protein isoelectric point values*.

e *Protein fold changes compared to the control (dry seeds). “+” up-regulated; “−” down-regulated after 24 h imbibition*.

f *Protein annotation of the best matching BLAST hit of the rice database in NCBI*.

g *Abbreviations for the rice genes encoding the identified proteins; To separate the same name of genes located on the same or different chromosomes, the genes were numbered though adding No. of chromosome in the abbreviations*.

### Meta-analysis of gene expression

**Figure 2:**
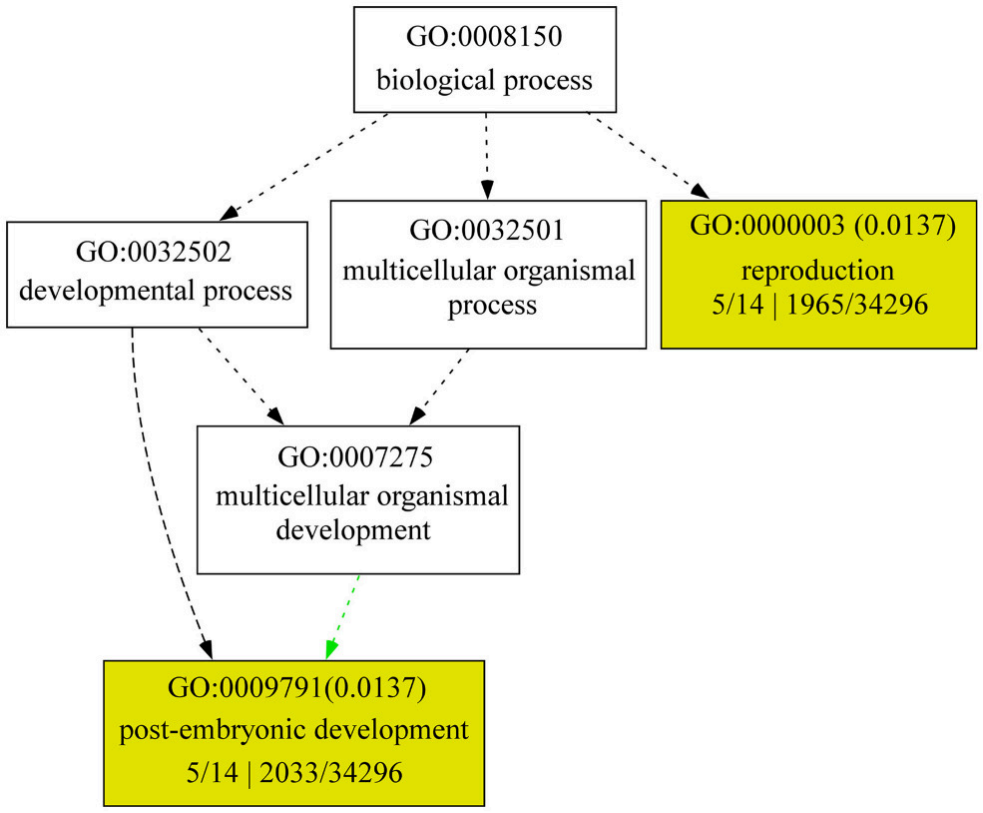
**GO enrichment analysis for changed proteins**. GO terms, such as “biological process,” “molecular function,” and “cellular component,” were identified using AGRIGO (http://bioinfo.cau.edu.cn/agriGO/index.php) with default significance levels (FDR < 0.05). 5/14 means gene number in input list; 1965/32496 and 2033/32496 means gene number in BG/Ref.

**Figure 3:**
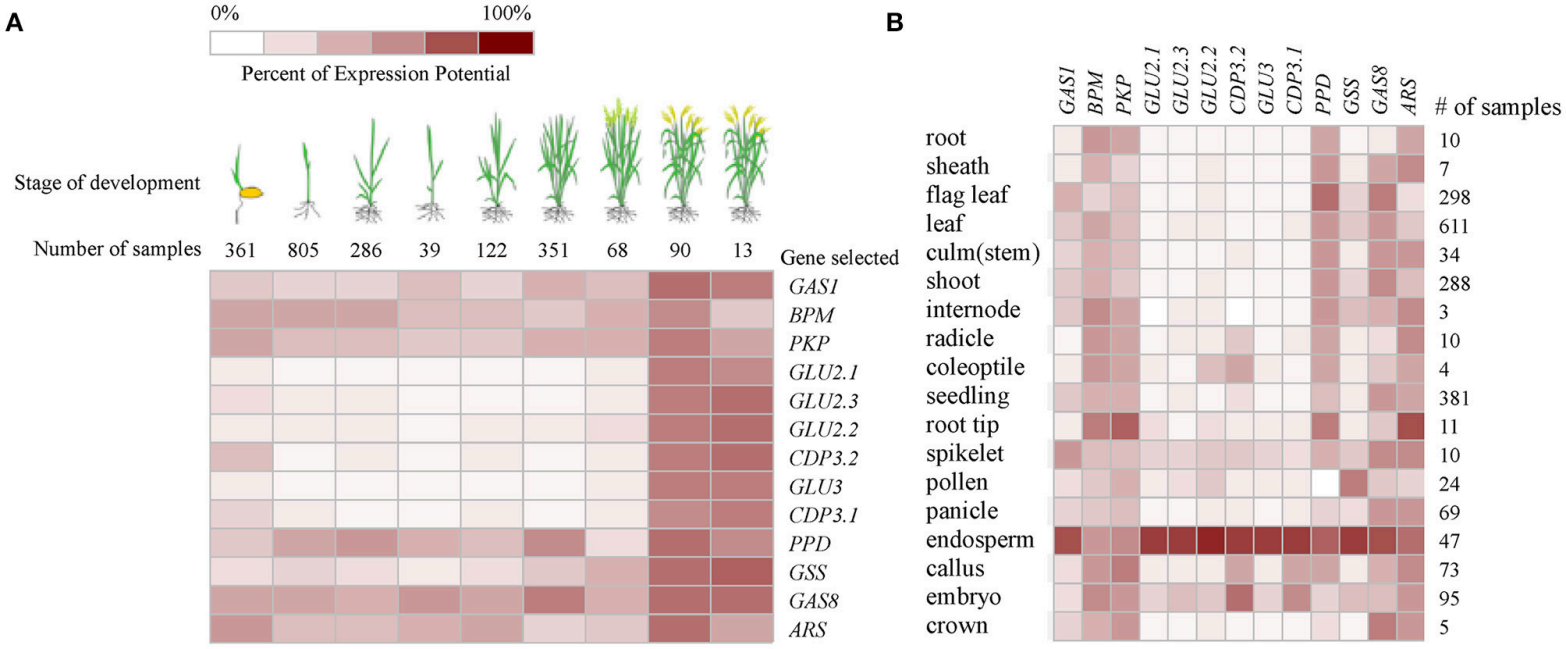
**Expression pattern of genes encoding the identified proteins in various developmental stages (A)** and tissues **(B)** of rice based on the publicly available microarray data (http://www.genevestigator.com).

Based on the publicly available microarray database, the genes encoding the identified proteins were summarized for an overview of the genes characteristics. Gene ontology (GO) analyses showed that five genes *GLU2.1, GLU2.2, GLU2.3, GLU3*, and *GAS8* involved in the processes of reproduction (GO:0000003) and post-embryonic development (GO:0009791) (Figure 2). The higher transcript abundances of five genes *BPM, PKP, PPD, GAS8*, and *ARS* were observed at all of the developmental stages and in various tissues (Figure 3). However, the medium transcript abundances of another eight genes *GAS1, GLU2.1, GLU2.2, GLU2.3, GLU3, CDP3.1, CDP3.2*, and *GSS* were observed from the germination to the early reproductive (flowering) stages, with sharp up-regulation during seed development (milk and dough) stages.

### Dynamic gene expression during seed imbibition

**Figure 4:**
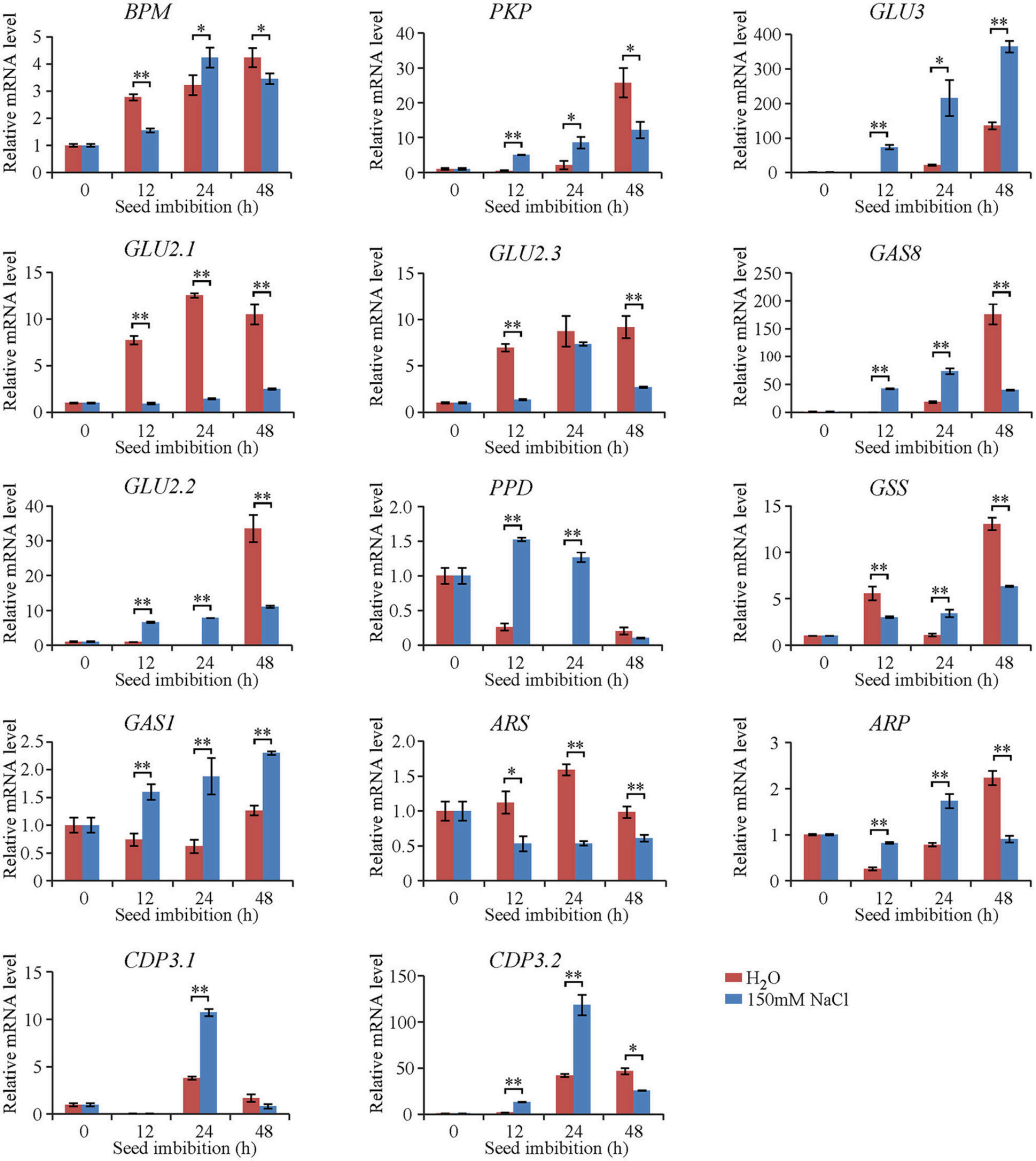
**Expression pattern of genes during seed imbibition under distilled water and salt conditions based on quantitative RT–PCR analysis**. The mRNA level of genes was normalized to that of 18S ribosomal RNA gene control. Each column represents means ± standard deviation. ^*^ and ^**^ indicated the significant difference at 5 and 1% levels between water and salt conditions, respectively.

To elucidate the characterization of genes encoding the identified proteins during seed imbibition, dynamic qRT-PCR analyses were conducted in Jiucaiqing after 12, 24, and 48 h imbibition in distilled water and 150 mM NaCl. The expression of eight genes such as *BPM, PKP, GLU3, GLU2.1, GLU2.3, GAS8, GLU2.2*, and *GSS* was increased during imbibition under both water and salt conditions in generally, while the expression of four genes such as *PPD, GAS1, ARS*, and *ARP* was stable (Figure 4). By comparison, the expression of nine genes, including *PKP, GAS1, GAS8, GLU2.2, GLU3, PPD, ARP, CDP3.1*, and *CDP3.2*, was significantly higher after 12 and 24 h imbibition under salt condition compared with water condition, suggesting the most genes encoding the identified proteins were earlier induced during imbibition by salt stress.

### Integrating proteomics data and QTLs for seed germination

**Figure 5:**
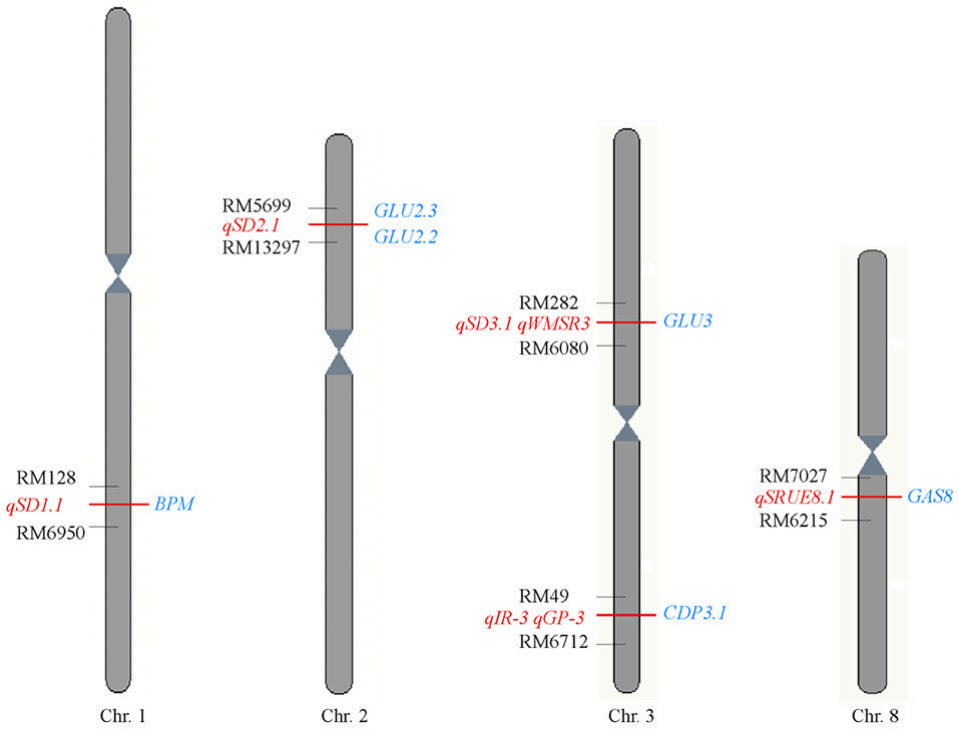
**Comparison of position between the identified proteins here with the previous QTLs for seed germination among Jiucaiqing/IR26 RILs population**. The proteins identified in this study are shown on the left (red), and the previously mapped QTLs are shown on the right (blue) (Wang et al., 2011, 2014; Cheng et al., 2013; Liu et al., 2014).

The genome regions of QTLs for seed germination in Jiucaiqing (Wang et al., 2011, 2014; Cheng et al., 2013; Liu et al., 2014) and genes encoding the identified proteins here were compared (Figure 5). Four genes, *BPM, GLU2.3, GLU2.2*, and *CDP3.2*, were near the regions of three QTLs for seed dormancy in Jiucaiqing. Two genes, *CDP3.2* and *GAS8*, were mapped near the regions of QTLs for seed reserve utilization. The *CDP3.1* gene was co-located in the region of *qIR-3* for imbibition rate in distilled water and *qGP-3* for germination percentage under salt condition.

### Role of a cupin domain containing protein

**Figure 6:**
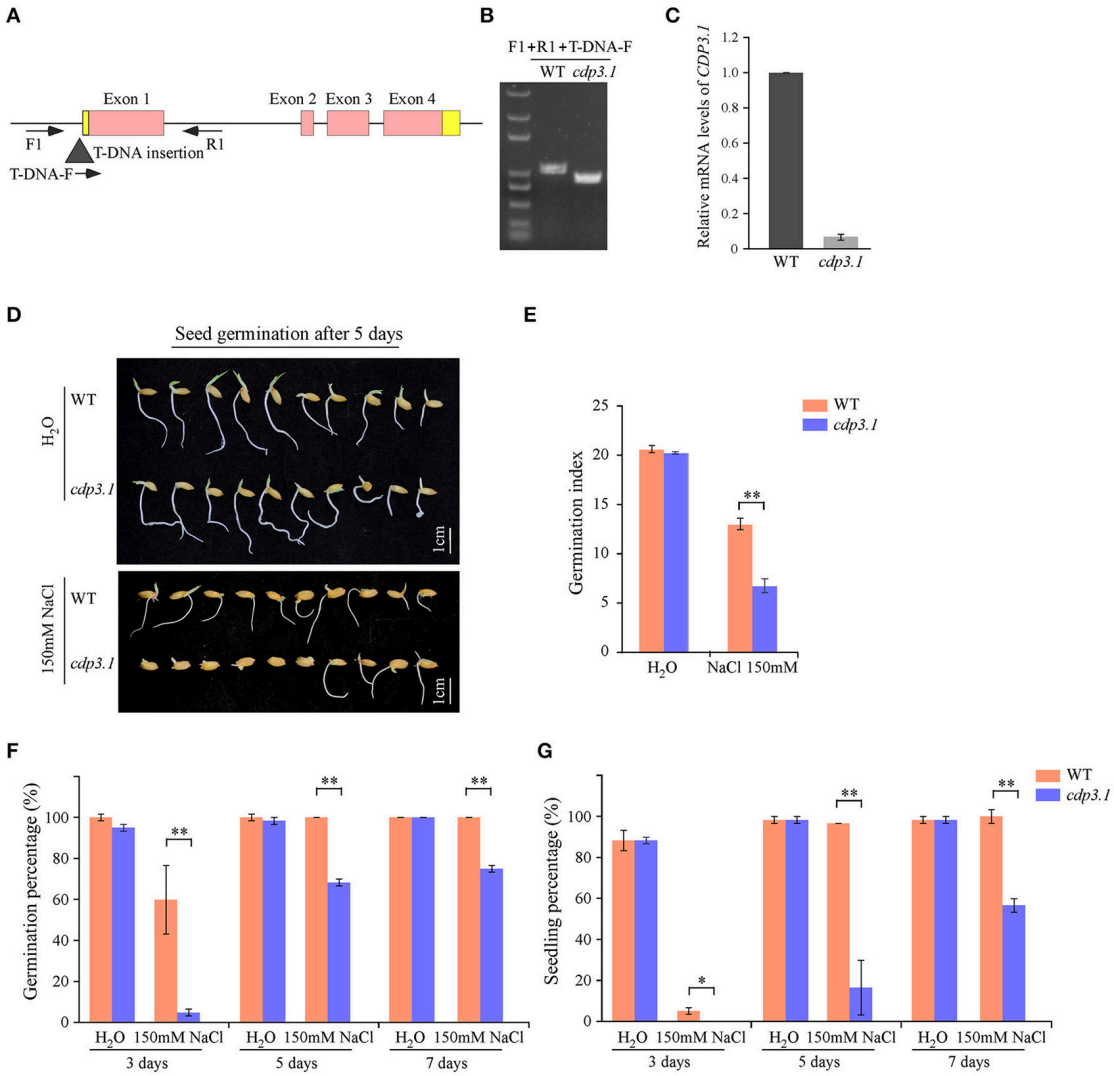
**Comparison of seed germination between wide type (WT) and *CDP3.1* mutant line (*cdp3.1*) under water and salt stress conditions. (A)** Gene structure of *CDP3.1* with a T-DNA insertion and the locations of the primers used for PCR analysis. Black triangle represents the T-DNA. Red and yellow boxes represent exons and UTR of *CDP3.1*, respectively. Solid lines represent introns. F, R, and T-DNA-F primers used for genotyping PCR. **(B)** PCR of genomic DNA from WT and *cdp3.1*. **(C)** Quantitative RT-PCR analysis of *CDP3.1* mRNA level from WT and *cdp3.1* seeds. The mRNA level of *CDP3.1* was normalized to that of 18S ribosomal RNA gene control. **(D)** Seed germination of WT and *cdp3.1* after 5 days under water and 150 mM NaCl conditions. **(E)** Germination index of WT and *cdp3.1* after 10 days under water and 150 mM NaCl conditions. **(F)** Germination percentage of WT and *cdp3.1* after 3, 5, and 7 days under water and 150 mM NaCl conditions. **(G)** Seedling percentage of WT and *cdp3.1* after 3, 5, and 7 days under water and 150 mM NaCl conditions. Each column represents means ± standard deviation. ^*^ and ^**^ indicated the significant difference at 5 and 1% levels, respectively.

**Figure 7:**
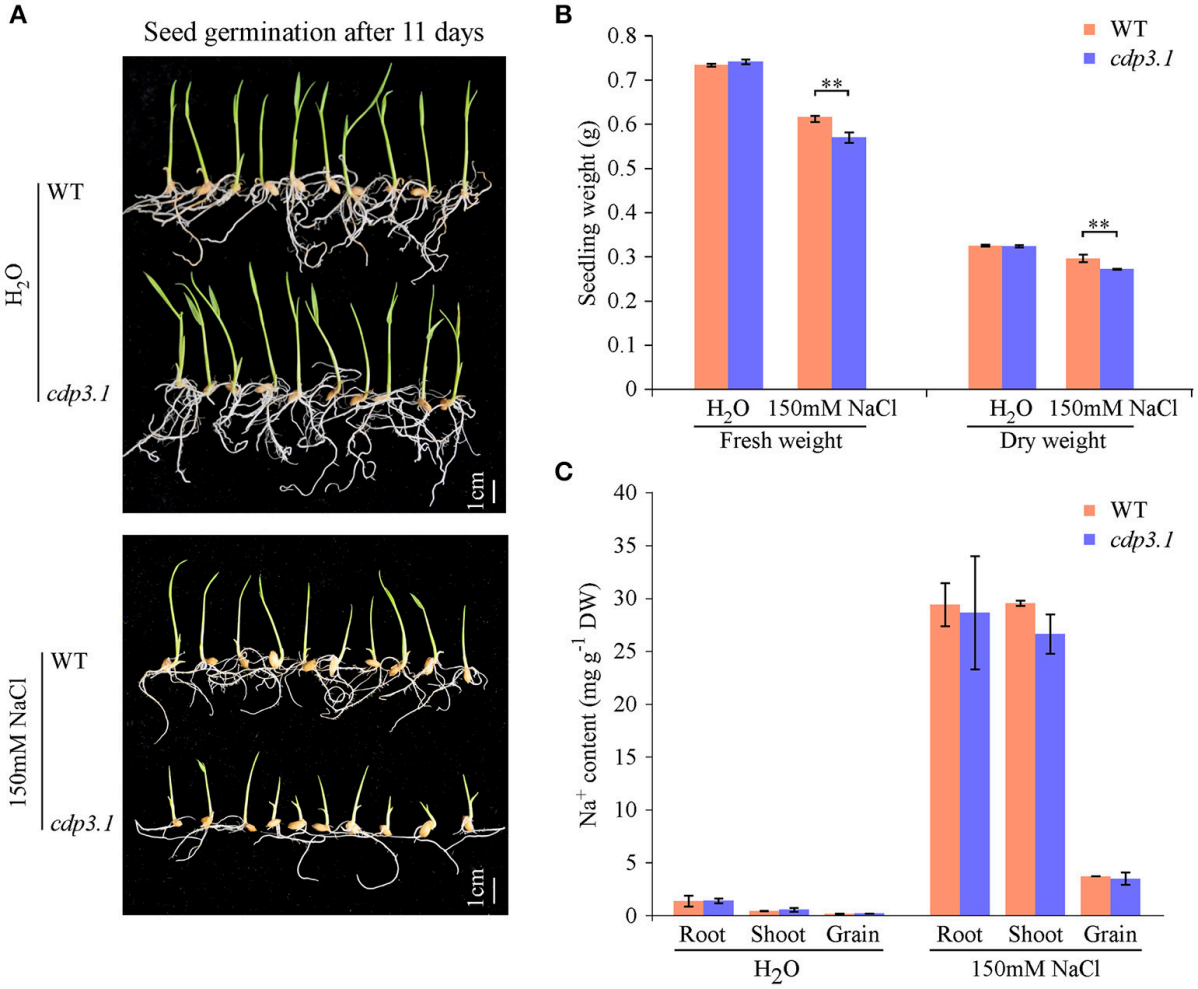
**Comparison of seedling growth and Na^+^ ion accumulation in tissues between wide type (WT) and *CDP3.1* mutant line (*cdp3.1*) under water and salt stress conditions. (A)** Seedling growth of WT and *cdp3.1* after 11 days under water and 150 mM NaCl conditions. **(B)** Fresh and dry seedling weight of WT and *cdp3.1* after 11 days under water and 150 mM NaCl conditions. **(C)** Accumulation of Na^+^ in grains, roots, and shoots of WT and *cdp3.1* after 11 days under water and 150 mM NaCl conditions. Each column represents means ± standard deviation. ^**^ indicated the significant difference at 1% level.

As described above, *CDP3.1* was near to the region of *qIR-3* and *qGP-3*, suggesting that *CDP3.1* plays important roles in seed germination. Therefore, one T-DNA insertion mutant was used to confirm the role of *CDP3.1* on seed germination in rice. The insertion of T-DNA in the promoter of *CDP3.1* gene was verified by PCR analysis (Figure 6A), and homozygous mutant line defective in *CDP3.1* was confirmed at the DNA and mRNA levels (Figures 6B,C). Homozygous *cdp3.1* mutant had an decrease in seed germination as compared to WT under salt stress (Figure 6D). There were no significant differences in GI after 10 days germination between WT and *cdp3.1* under water condition. Similarly, no significant differences were observed in GP and SP after 3, 5 and 7 days germination. However, those traits were significantly decreased in *cdp3.1* compared to WT under salt stress (Figures 6E–G). The dry and fresh weight of seedlings were significantly decreased in *cdp3.1* as compared to WT under salt stress (Figures 7A,B), while the level of Na^+^ was not significantly accumulated in developing seeds, roots, and shoots of *cdp3.1* (Figure 7C).

## Discussion

The influx of water into the cells of dry seeds during Phase I of imbibition is a physical process. Therefore, similar results were observed at the first ~24 h in both distilled water (Cheng et al., 2017) and salt conditions in Jiucaiqing. Our previous study indicated that Phase II began at ~24 to 84 h in distilled water (Cheng et al., 2017). However, our current study indicated that the Phase II imbibition was prolonged to 96 h under salt stress. These results may explain, at least in part, why seeds had slower germination speed in salt solution than in distilled water due to a prolonged Phase II. Indeed, the maximum germination was attained after 60 h imbibition in distilled water (Cheng et al., 2017), but after 96 h imbibition under salt condition. In this study, we are mainly to identify genes involved in seed imbibition under salt stress. Therefore, the dry seeds of Jiucaiqing were used as control to perform the comparative proteomic assays in this study.

In the current study, the differences for expressed proteins at a change >2.0-fold and *P* < 0.01 were used as screening criteria (Cheng et al., 2017). We identified 14 proteins involved in the early seed imbibition (24 h) under salt stress. By comparison, the glucose-1-phosphate adenylyltransferase large subunit protein (GAS1 and GAS8) were identified under salt stress as well as in distilled water (Cheng et al., 2017), suggesting that GAS proteins are required for seed germination under various conditions. However, other proteins detected under salt stress may have specific functions. Furthermore, six proteins had been detected involved in seed germination in previous reports, including 2,3-bisphosphoglycerate-independent phosphoglycerate mutase (Graña et al., 1993), cupin domain containing protein (Lapik and Kaufman, 2003), glutelin (Yang et al., 2007), 60S ribosomal protein (Rajjou et al., 2008), pyruvate phosphoglycerate dikinase (Weitbrecht et al., 2011), and granule-bound starch synthase I (Liu et al., 2015). However, the argininosuccinate synthase and pyruvate phosphate dikinase protein were first time found to be involved in seed germination under salt stress. Six genes encoding the proteins identified here are near the regions of QTLs for seed dormancy, seed reserve utilization, seed imbibition and seed germination in our previous studies (Wang et al., 2011, 2014; Cheng et al., 2013; Liu et al., 2014). It indicates that these genes might be important for seed germination.

To further evaluate the function of the genes, we categorized them based on their known functions using GO classifications. We found that glucose-1-phosphate adenylyltransferase large subunit protein and glutelin are significantly associated with the processes of reproduction and post-embryonic development. Glucose-1-phosphate adenylyltransferase is an enzyme that catalyzes the formation of adenosine triphosphate (ATP) to adenosine diphosphate (ADP) and vice versa. In previous study, seed dormancy QTL *qSdn-1* was associated with glucose-1-phosphate adenylyltransferase large subunit 1 (Qin et al., 2010). The cereals store reserve proteins (globulin *and glutelins*) provide nitrogen for seed germination and early seedling growth (Kishimoto et al., 2001; Yano et al., 2001). The storage reserve proteins glutelin (GLU2.1, GLU2.2, and GLU3) are accumulated during seed development and were found to be down-regulated during seed imbibition in this study. Similarly, Yang et al. (2007) indicated that the glutelin was down-regulated at the late stage of germination Phase II (48 h). However, we also found that the glutelin GLU2.3 was up-regulated during seed imbibition under salt stress. The function of glutelin GLU2.3 during seed germination need to be further researched.

The changes of proteins related to energy metabolism, e.g., pyruvate phosphate dikinase, 2,3-bisphosphoglycerate-independent phosphoglycerate mutase and phosphoglycerate kinase protein, were identified in the early Phase II (24h) of imbibition under salt stress in rice. Pyruvate phosphate dikinase is one of the main limiting enzymes in the process of glycolysis that catalyzes the inter-conversion of ATP and pyruvate with adenine monophosphate (AMP) and phosphoenolpyruvate (PEP; Evans and Wood, 1968). 2,3-biphosphoglycerate-independent phosphoglycerate mutase is a key enzymatic activity in glycolysis and catalyses the reversible interconversion of 3-phosphoglycerate to 2-phosphoglycerate (Zhao and Assmann, 2011). Phosphoglycerate kinase is an enzyme that catalyzes the reversible transfer of a phosphate group from 1,3-bisphosphoglycerate (1,3-BPG) to ADP producing 3-phosphoglycerate (3-PG) and ATP (Fermo et al., 2012). These results suggest that those enzymes play important roles in energy metabolism during early seed imbibition under salt stress in rice. An increased ATP requirement might be required for Na^+^ extrusion and maintaining the membrane potential. The link between the energy metabolism and avoidance mechanisms of salt stress would be further investigated.

In order to clarify the characterization of these gene expression during seed imbibition, a quantitative PCR-based gene expression experiment was conducted. By comparison, the most of identified proteins were down-regulated after 24 h imbibition, while the mRNA levels were up-regulated. Only five proteins (ARS, ARP, CDP3.1, CDP3.2, and GLU2.3) behaved the similar transcript and protein expression patterns during seed germination. The reason might be that the catabolism of these storage proteins at the early imbibition stage is necessary for seed germination, while the increase of mRNA level at the end of germination for *de novo* protein synthesis is essential for vigorous seedling growth. Interestingly, the expression of the majority genes encoding the identified proteins was significantly higher under salt condition compared with water condition in the early imbibition stage (12 and 24 h). It suggests that the earlier induction of these genes might be important for successful seed germination under salt condition. More experiments should be performed to confirm this hypothesis.

It has been estimated previously that cupins had at least 18 different functional subclasses (Dunwell et al., 2001), which include enzymatic functions like hydrolases, dioxygenases, decarboxylases, isomerases, and epimerases non-enzymatic functions such as seed storage, binding to auxin, and nuclear transcription factors (Dunwell et al., 2001, 2004). In this study, we found that the induction of cupin domain containing protein CDP3.1 (globulin) benefits seed germination and seedling growth under salt stress. However, the improvement of salinity tolerance by CDP3.1 is not by reducing Na^+^ uptake of seedlings, indicating it may play roles on salinity tolerance by other mechanism. For example, the accumulation of storage proteins are associated with desiccation tolerance (Bäumlein et al., 1995), and they also be used as a source of energy for the germinating embryo (Dunwell et al., 2000). In this study, the accumulation of *CDP3.1* might improve seed quality during seed development, and then provide more energy for seed germination under salt stress. The function of *CDP3.1* on seed germination should be confirmed using more transgenic lines or mutant lines; the construction of RNAi transgenic lines and CRISPR/Cas9 mutant lines for *CDP3.1* is being investigated in rice.

In summary, imbibition is a critical process during seed germination. To detect proteins which contribute to seed germination under salt stress, the changes of proteomic in 24 h imbibed seeds under salt stress were investigated in this study. We identified 14 proteins involved in seed imbibition under salt stress in rice. The most of proteins were down-regulated by imbibition; it appears that the early imbibition process is mediated by protein catabolism rather than by *de novo* protein synthesis. The majority of these proteins were involved in energy supply and storage protein. In particular, our results confirmed the role of cupin globulin protein CDP3.1 in governing seed germination under salt stress. The function of these identified proteins in seed germination need to be studied in the future.

## Author contributions

Conceived and designed the experiments: ZW and HZ. Performed the experiments: EX, MC, and CZ. Analyzed the data: HH and YC. Wrote the paper: ZW.

### Conflict of interest statement

The authors declare that the research was conducted in the absence of any commercial or financial relationships that could be construed as a potential conflict of interest.

## Supplementary material

Supplementary Table 1 **The primer pairs of genes for quantitative RT-PCR**.

Supplementary Table 2 **Peptide sequences identified by MS-MS sequencing and corresponding to proteins identified in rice of this work**.

Supplementary Figure 1 **2-D analysis of rice proteome during seed germination under 150 mM NaCl condition**. 2-D gel profiles of total proteins from dry seeds **(A)** and 24 h imbibed seeds **(B)**, and the proteins statistically significant differences (*P* < 0.05) at least 1.5-fold between dry and 24 h imbibed seeds **(C)**. An equal amount (100 μg) of total protein extracts was loaded in each gel.

Supplementary Figure 2 **Magnified views of the gels showing down- and up-regulated proteins statistically significant differences (*P* < 0.01) at least 2-fold between 24 h imbibed and dry seeds under 150 mM NaCl condition**. Proteins eluted from these spots were successfully identified by MALDI-TOF-MS (see Table 1).
