# Supplementary material for: Proteomic Analysis Reveals Proteins Involved in Seed Imbibition under Salt Stress in Rice
Source: Front Plant Sci. 2017 Jan 5;7:2006. doi: 10.3389/fpls.2016.02006 (PMC5213780; doi:10.3389/fpls.2016.02006)
Supplement: Supplementary Table 1 — The primer pairs of genes for quantitative RT-PCR. [file Table1.DOCX]

**Supplemental Tables**

**Supplementary Table 1** The primer pairs of genes for quantitative RT-PCR

| Gene | Forwad primer (5’–3’) | Reverse primer (5’–3’) |
| --- | --- | --- |
| 18S ribosomal | CTACGTCCCTGCCCTTTGTACA | ACACTTCACCGGACCATTCAA |
| LOC_Os03g57960 | ACACACACAGCTCCAAGTGGTTC | GTTGGAGAAGTACGGACTCTTGC |
| LOC_Os01g60190 | CCAGCCACTTCTCGACAAGA | GAAGACCGGGACCTCCAATAG |
| LOC_Os02g07260 | TTCACTTATTGAGAAGGCGAAGG | TAGTGTTGCAGGTGTCCAATGC |
| LOC_Os05g33570 | ATCTGTCTCAGGGTATCCTCCAG | GAAACATAGTCCAGCCCAGCC |
| LOC_Os08g25734 | AGCTGACTACTACGAGACTGAAGCAG | GCCACTTTTGATAAAGTATCCATCAG |
| LOC_Os12g13320 | AATGCACCTTCAAAACCTGAGTAT | ACCATATCAATGCGCCCAATAC |
| LOC_Os01g44220 | AGGCAAGGTCCCAATTGGTATAG | TTGATGGTTGCATTCTTCAGGAT |
| LOC_Os02g14600 | ACCCAAATGCCATGGTAAACC | TTGGATTTGAGTACTCTTGGTGGAT |
| LOC_Os02g16820 | AAACTCCATGGTGAGCCACCT | GCCTAGGTAGGTCTGTTGTTGATATC |
| LOC_Os02g25640 | CACAATGCTGTCGACAGTCAGAT | TCAGCATACTGTTGCGGCC |
| LOC_Os03g21790 | ACATCGGCGTCTATCTCGTCA | TTCGGGAACACGACCTCGA |
| LOC_Os03g31360 | GGAAGGATGCTCATATATTGCATT | CACTCACAGATATGTCTTGGTAGCTC |
| LOC_Os06g04200 | GTACGAGGAGATGGTCAGGAACT | CACATTCTCCCAGTTCTTCGC |
| LOC_Os11g04070 | CGAATACTCATACCCACATGCTGAT | TTCCTCCTTGGAAGCAGCTG |
