# Supplementary material for: Proteomic Analysis Reveals Proteins Involved in Seed Imbibition under Salt Stress in Rice
Source: Front Plant Sci. 2017 Jan 5;7:2006. doi: 10.3389/fpls.2016.02006 (PMC5213780; doi:10.3389/fpls.2016.02006)
Supplement: Supplementary Table 2 — Peptide sequences identified by MS-MS sequencing and corresponding to proteins identified in rice of this work. [file Table2.DOCX]

**Supplemental Tables**

**Supplementary Table 2** Peptide sequences identified by MS-MS sequencing and corresponding to proteins identified in rice of this work

| Spots ^a^ | Protein Name | Calc. Mass | Obsrv. Mass | ± da | ± ppm | Start Seq. | End Seq. | Sequence | Ion Score | C. I. % |
| --- | --- | --- | --- | --- | --- | --- | --- | --- | --- | --- |
| 488 | Glucose-1-phosphate adenylyltransferase large subunit | 839.408 | 839.3992 | -0.0088 | -10 | 219 | 225 | EMVAYAR |  |  |
|  |  | 865.4778 | 865.476 | -0.0018 | -2 | 93 | 99 | IFDTTLR |  |  |
|  |  | 939.4968 | 939.4904 | -0.0064 | -7 | 612 | 620 | MSSFVGAIK |  |  |
|  |  | 991.4843 | 991.4792 | -0.0051 | -5 | 85 | 92 | IDDPNYVR |  |  |
|  |  | 1030.543 | 1030.539 | -0.0036 | -3 | 77 | 84 | RPEYVPNR |  |  |
|  |  | 1047.583 | 1047.582 | -0.0014 | -1 | 421 | 430 | ANEFGIVLGK |  |  |
|  |  | 1145.559 | 1145.563 | 0.0043 | 4 | 175 | 184 | DIDAAWEAVR | 54 | 99.68 |
|  |  | 1183.705 | 1183.683 | -0.0219 | -19 | 208 | 218 | KTPEQVVAIAK | 12 | 0 |
|  |  | 1328.717 | 1328.733 | 0.0164 | 12 | 320 | 331 | QLEVTINGIGER |  |  |
|  |  | 1447.746 | 1447.751 | 0.0051 | 4 | 332 | 345 | AGNASLEEVVMAIK |  |  |
|  |  | 1452.842 | 1452.853 | 0.0105 | 7 | 535 | 547 | AVDDIIQIPTVLR | 28 | 0 |
|  |  | 1492.747 | 1492.755 | 0.0086 | 6 | 520 | 534 | IACAVGTGPVDAAYK |  |  |
|  |  | 1635.87 | 1635.885 | 0.0147 | 9 | 587 | 603 | AFSGSGAALDIVVSSVR |  |  |
|  |  | 1649.838 | 1649.851 | 0.0131 | 8 | 406 | 420 | GTYEIISPDDIGLTR | 25 | 0 |
|  |  | 1735.759 | 1735.787 | 0.0281 | 16 | 226 | 241 | SLGCPDVEFSPEDAGR | 49 | 99.047 |
|  |  | 1940.997 | 1941.021 | 0.0241 | 12 | 404 | 420 | YKGTYEIISPDDIGLTR |  |  |
|  |  | 1960.917 | 1960.974 | 0.0566 | 29 | 548 | 565 | EYSMTSVTEGIDAIATTR |  |  |
|  |  | 2003.009 | 2003.03 | 0.0203 | 10 | 77 | 92 | RPEYVPNRIDDPNYVR |  |  |
|  |  | 2257.135 | 2257.175 | 0.0398 | 18 | 124 | 145 | LGVDIIEAGFPASSPDDLDAVR | 44 | 97.103 |
|  |  | 2576.314 | 2576.292 | -0.0217 | -8 | 146 | 170 | SIAIEVGNTPVGEDGHVPVICGLSR |  |  |
| 501 | 2,3-bisphosphoglycerate-independent phosphoglycerate mutase | 896.4724 | 896.4712 | -0.0012 | -1 | 341 | 348 | TSGEYLVK |  |  |
|  |  | 910.5105 | 910.5035 | -0.007 | -8 | 163 | 170 | VHILTDGR |  |  |
|  |  | 915.4062 | 915.3971 | -0.0091 | -10 | 207 | 213 | MYVTMDR |  |  |
|  |  | 1005.536 | 1005.535 | -0.0017 | -2 | 237 | 245 | FQNAVEAVK |  |  |
|  |  | 1042.487 | 1042.485 | -0.0022 | -2 | 353 | 361 | TFACSETVK |  |  |
|  |  | 1323.633 | 1323.665 | 0.032 | 24 | 214 | 223 | YENDWDVVKR | 34 | 66.447 |
|  |  | 1330.704 | 1330.693 | -0.0108 | -8 | 110 | 120 | IFEGEGFKYIK |  |  |
|  |  | 1348.722 | 1348.72 | -0.0022 | -2 | 412 | 423 | DAILSGKFDQVR | 80 | 100 |
|  |  | 1481.707 | 1481.716 | 0.0083 | 6 | 362 | 373 | FGHVTFFWNGNR |  |  |
|  |  | 1482.737 | 1482.699 | -0.0384 | -26 | 349 | 361 | NGIRTFACSETVK |  |  |
|  |  | 1636.869 | 1636.877 | 0.008 | 5 | 327 | 340 | LPSHYLVSPPEIER | 46 | 97.834 |
|  |  | 2079.915 | 2079.92 | 0.0052 | 3 | 207 | 222 | MYVTMDRYENDWDVVK |  |  |
|  |  | 2397.154 | 2397.069 | -0.0845 | -35 | 424 | 446 | VNLPNGDMVGHTGDIEA |  |  |
| 633 | Glucose-1-phosphate adenylyltransferase large subunit | 928.5714 | 928.5633 | -0.0081 | -9 | 303 | 310 | DVLLDILK |  |  |
|  |  | 952.4887 | 952.4874 | -0.0013 | -1 | 372 | 379 | TPFFTSPR | 51 | 99.235 |
|  |  | 1027.578 | 1027.575 | -0.0035 | -3 | 450 | 459 | VPIGIGENTK |  |  |
|  |  | 1086.631 | 1086.635 | 0.0043 | 4 | 380 | 388 | YLPPARLEK |  |  |
|  |  | 1106.508 | 1106.516 | 0.0077 | 7 | 462 | 470 | NCIIDMNAR | 19 | 0 |
|  |  | 1140.51 | 1140.516 | 0.006 | 5 | 43 | 53 | CFGDGVTGTAR | 20 | 0 |
|  |  | 1155.554 | 1155.557 | 0.0028 | 2 | 215 | 223 | MNYMELVQK |  |  |
|  |  | 1247.62 | 1247.634 | 0.0136 | 11 | 103 | 114 | ATPAVPVGGCYR | 58 | 99.831 |
|  |  | 1373.63 | 1373.704 | 0.0734 | 53 | 2 | 13 | PLDTNACAQPMR |  |  |
|  |  | 1375.693 | 1375.711 | 0.0174 | 13 | 460 | 470 | IRNCIIDMNAR |  |  |
|  |  | 1473.711 | 1473.732 | 0.0213 | 14 | 274 | 286 | VDTSFLSYAIDDK |  |  |
|  |  | 1529.731 | 1529.743 | 0.0121 | 8 | 2 | 14 | PLDTNACAQPMRR |  |  |
|  |  | 1627.826 | 1627.861 | 0.0343 | 21 | 131 | 144 | IFVMTQFNSASLNR | 31 | 17.803 |
|  |  | 1645.833 | 1645.87 | 0.0364 | 22 | 313 | 326 | YAHLQDFGSEILPR | 101 | 100 |
|  |  | 1645.902 | 1645.87 | -0.0324 | -20 | 289 | 302 | YPYIASMGIYVLKK |  |  |
|  |  | 1677.848 | 1677.804 | -0.0442 | -26 | 350 | 364 | SFFDANLALTEQPPK |  |  |
|  |  | 1729.864 | 1729.86 | -0.0045 | -3 | 274 | 288 | VDTSFLSYAIDDKQK |  |  |
|  |  | 1789.829 | 1789.874 | 0.0454 | 25 | 54 | 68 | CVFTSDADRDTPHLR |  |  |
|  |  | 1822.883 | 1822.924 | 0.041 | 22 | 115 | 130 | LIDIPMSNCFNSGINK |  |  |
|  |  | 1831.857 | 1831.897 | 0.0394 | 22 | 335 | 349 | ACVFTEYWEDIGTIK | 71 | 99.992 |
|  |  | 1860.96 | 1861.005 | 0.0447 | 24 | 311 | 326 | SKYAHLQDFGSEILPR |  |  |
|  |  | 1933.994 | 1934.01 | 0.016 | 8 | 257 | 273 | VIQFLEKPEGADLESMK |  |  |
|  |  | 2055.94 | 2056.003 | 0.0633 | 31 | 224 | 242 | HVDDNADITISCAPIDGSR | 108 | 100 |
|  |  | 2604.266 | 2604.276 | 0.0103 | 4 | 350 | 371 | SFFDANLALTEQPPKFEFYDPK |  |  |
|  |  | 2632.264 | 2632.386 | 0.1218 | 46 | 474 | 496 | NVIIANTQGVQESDHPEEGYYIR | 110 | 100 |
|  |  | 2831.469 | 2831.506 | 0.0373 | 13 | 75 | 102 | NYADASHVSAVILGGGTGVQLFPLTSTR | |  |
|  |  | 2981.434 | 2981.507 | 0.073 | 24 | 391 | 417 | IKDAIISDGCSFSECTIEHSVIGISSR |  |  |
| 706 | Glucose-1-phosphate adenylyltransferase large subunit | 854.444 | 854.4254 | -0.0186 | -22 | 206 | 213 | ATAFGLMK |  |  |
|  |  | 900.5261 | 900.4778 | -0.0483 | -54 | 428 | 435 | AIIDKNAR |  |  |
|  |  | 972.4673 | 972.4741 | 0.0068 | 7 | 454 | 461 | ETDGYFIK |  |  |
|  |  | 1002.576 | 1002.577 | 0.0005 | 0 | 266 | 273 | NVMLQLLR | 10 | 0 |
|  |  | 1010.545 | 1010.545 | -0.0001 | 0 | 205 | 213 | RATAFGLMK |  |  |
|  |  | 1032.547 | 1032.552 | 0.0052 | 5 | 336 | 344 | SAPIYTQPR | 14 | 0 |
|  |  | 1060.604 | 1060.586 | -0.0174 | -16 | 220 | 228 | IVEFAEKPK |  |  |
|  |  | 1256.711 | 1256.718 | 0.0069 | 5 | 70 | 81 | AKPAVPLGANYR | 23 | 0 |
|  |  | 1341.712 | 1341.723 | 0.0106 | 8 | 442 | 453 | IINVDNVQEAAR | 64 | 99.966 |
|  |  | 1361.677 | 1361.69 | 0.0131 | 10 | 13 | 24 | SNVASEQQQSKR |  |  |
|  |  | 1370.674 | 1370.689 | 0.0145 | 11 | 325 | 335 | KPVPDFSFYDR | 74 | 99.996 |
|  |  | 1615.905 | 1615.911 | 0.0052 | 3 | 220 | 233 | IVEFAEKPKGEQLK | 1 | 0 |
|  |  | 1681.814 | 1681.81 | -0.0033 | -2 | 234 | 248 | AMMVDTTILGLDDVR | 23 | 0 |
|  |  | 1700.918 | 1700.935 | 0.0176 | 10 | 44 | 61 | SVDESVLGIILGGGAGTR | 104 | 100 |
|  |  | 1889.953 | 1889.925 | -0.0277 | -15 | 351 | 368 | VLDADVTDSVIGEGCVIK |  |  |
|  |  | 1906.906 | 1906.917 | 0.0105 | 6 | 189 | 205 | ETDSDITVAALPMDEKR |  |  |
|  |  | 2295.162 | 2295.176 | 0.0144 | 6 | 442 | 461 | IINVDNVQEAARETDGYFIK |  |  |
|  |  | 2423.13 | 2423.16 | 0.0308 | 13 | 274 | 296 | EQFPGANDFGSEVIPGATNIGMR |  |  |
| 748 | Argininosuccinate synthase | 819.4723 | 819.4722 | -0.0001 | 0 | 466 | 472 | LYGLPTR |  |  |
|  |  | 833.4304 | 833.4324 | 0.002 | 2 | 398 | 403 | WFDPLR |  |  |
|  |  | 876.4244 | 876.4238 | -0.0006 | -1 | 338 | 344 | IDMVENR |  |  |
|  |  | 885.4465 | 885.4495 | 0.003 | 3 | 437 | 443 | SPYSLYR |  |  |
|  |  | 1013.541 | 1013.542 | 0.001 | 1 | 436 | 443 | KSPYSLYR |  |  |
|  |  | 1041.536 | 1041.541 | 0.0041 | 4 | 389 | 397 | YAELVYAGR | 38 | 85.654 |
|  |  | 1193.664 | 1193.625 | -0.0386 | -32 | 425 | 435 | LYKGSVNVASR |  |  |
|  |  | 1519.867 | 1519.834 | -0.0324 | -21 | 168 | 181 | YLLGTSMARPVIAK |  |  |
|  |  | 1584.831 | 1584.854 | 0.0234 | 15 | 209 | 221 | FELTFYALNPELK |  |  |
|  |  | 1592.81 | 1592.827 | 0.0167 | 10 | 352 | 367 | GVYETPGGTIMAAAVR | 64 | 99.965 |
|  |  | 1795.85 | 1795.884 | 0.0342 | 19 | 228 | 242 | EWDITGREDAIEYAK |  |  |
|  |  | 1851.938 | 1851.962 | 0.0236 | 13 | 350 | 367 | SRGVYETPGGTIMAAAVR |  |  |
|  |  | 1878.927 | 1878.957 | 0.0306 | 16 | 368 | 382 | ELESLTLDRETMQWK |  |  |
|  |  | 2061 | 2061.041 | 0.0407 | 20 | 144 | 159 | DLKEEFVSEYIYPCLR | 18 | 0 |
|  |  | 2254.15 | 2254.176 | 0.0251 | 11 | 203 | 221 | GNDQVRFELTFYALNPELK |  |  |
|  |  | 2504.121 | 2504.166 | 0.0442 | 18 | 444 | 465 | EDISSFENGEIYNQADAEGFIR | 74 | 99.996 |
| 840 | Cupin domain containing protein | 877.489 | 877.4915 | 0.0025 | 3 | 435 | 443 | LVFGGSAAR |  |  |
|  |  | 925.5465 | 925.5484 | 0.0019 | 2 | 96 | 104 | VAVLEAAPR |  |  |
|  |  | 953.4622 | 953.4699 | 0.0077 | 8 | 135 | 141 | RESFCVR |  |  |
|  |  | 1005.584 | 1005.588 | 0.0043 | 4 | 434 | 443 | KLVFGGSAAR |  |  |
|  |  | 1084.636 | 1084.649 | 0.0128 | 12 | 122 | 131 | EGEGVIVLLR | 22 | 0 |
|  |  | 1150.528 | 1150.547 | 0.019 | 17 | 265 | 274 | SPYFSNNHGK |  |  |
|  |  | 1178.605 | 1178.617 | 0.0121 | 10 | 212 | 220 | EELEKVFER |  |  |
|  |  | 1223.584 | 1223.605 | 0.0209 | 17 | 301 | 311 | GSMIAPNYNTR | 14 | 0 |
|  |  | 1239.568 | 1239.586 | 0.0187 | 15 | 275 | 284 | LFELTGDECR | 45 | 97.613 |
|  |  | 1239.579 | 1239.586 | 0.0076 | 6 | 301 | 311 | GSMIAPNYNTR |  |  |
|  |  | 1326.774 | 1326.751 | -0.023 | -17 | 289 | 300 | LDLQIGLANITR |  |  |
|  |  | 1350.81 | 1350.83 | 0.0191 | 14 | 448 | 459 | VLAAQPEQILLR | 62 | 99.959 |
|  |  | 1529.744 | 1529.772 | 0.0277 | 18 | 223 | 236 | EGGEITTAPEEQIR | 104 | 100 |
|  |  | 1564.776 | 1564.802 | 0.0265 | 17 | 81 | 95 | FPDEQVVGAAVGGYR | 119 | 100 |
|  |  | 1813.904 | 1813.938 | 0.0338 | 19 | 221 | 236 | QREGGEITTAPEEQIR |  |  |
|  |  | 1822.018 | 1822.054 | 0.036 | 20 | 444 | 459 | EADRVLAAQPEQILLR | 18 | 0 |
|  |  | 1884.874 | 1884.93 | 0.0566 | 30 | 49 | 65 | HGGEGGRPYHLGEESFR |  |  |
|  |  | 1890.919 | 1890.954 | 0.0347 | 18 | 245 | 264 | GGGGGSGSEWEIKPSSLTGK |  |  |
|  |  | 1986.998 | 1987.048 | 0.0501 | 25 | 223 | 240 | EGGEITTAPEEQIRELSK |  |  |
|  |  | 2028.97 | 2028.977 | 0.0066 | 3 | 105 | 121 | AFLQPSHYDADEVFYVK |  |  |
|  |  | 2218.081 | 2218.097 | 0.0164 | 7 | 142 | 163 | EGDAMVIPAGAIVYSANTHSSK |  |  |
| 870 | Granule-bound starch synthase I | 856.525 | 856.5202 | -0.0048 | -6 | 22 | 29 | SAPLSLLR |  |  |
|  |  | 993.5284 | 993.5073 | -0.0211 | -21 | 445 | 452 | LLKSMEEK |  |  |
|  |  | 1107.492 | 1107.534 | 0.0416 | 38 | 336 | 344 | GCELDNIMR |  |  |
|  |  | 1349.721 | 1349.705 | -0.0164 | -12 | 211 | 221 | ILNLNNNPYFK |  |  |
|  |  | 1350.672 | 1350.681 | 0.0088 | 7 | 550 | 561 | VVGTPAYEEMVR |  |  |
|  |  | 1368.712 | 1368.716 | 0.0044 | 3 | 386 | 398 | EALQAEAGLPVDR |  |  |
|  |  | 1564.769 | 1564.779 | 0.0102 | 7 | 259 | 271 | VAFCIHNISYQGR |  |  |
|  |  | 1729.807 | 1729.835 | 0.0282 | 16 | 272 | 285 | FAFEDYPELNLSER | 19 | 0 |
|  |  | 1794.971 | 1794.905 | -0.0663 | -37 | 382 | 398 | ALNKEALQAEAGLPVDR |  |  |
|  |  | 1804.803 | 1804.832 | 0.0292 | 16 | 288 | 303 | SSFDFIDGYDTPVEGR | 42 | 95.016 |
| 959 | Phosphoglycerate kinase protein | 819.4094 | 819.3848 | -0.0246 | -30 | 241 | 247 | SLVEEDK |  |  |
|  |  | 820.3835 | 820.3602 | -0.0233 | -28 | 130 | 136 | NDPEFAK |  |  |
|  |  | 1000.506 | 1000.463 | -0.0432 | -43 | 156 | 165 | AHASTEGVTK |  |  |
|  |  | 1072.651 | 1072.651 | -0.0003 | 0 | 75 | 83 | YSLKPLVPR | 20 | 0 |
|  |  | 1074.631 | 1074.635 | 0.004 | 4 | 192 | 202 | KPFAAIVGGSK |  |  |
|  |  | 1117.585 | 1117.592 | 0.0069 | 6 | 6 | 16 | SVGTLGEADLR | 43 | 95.974 |
|  |  | 1190.737 | 1190.742 | 0.005 | 4 | 59 | 69 | VILASHLGRPK |  |  |
|  |  | 1388.742 | 1388.737 | -0.0047 | -3 | 179 | 191 | ELDYLVGAVANPK |  |  |
|  |  | 1545.812 | 1545.811 | -0.0005 | 0 | 351 | 367 | GATTIIGGGDSVAAVEK |  |  |
|  |  | 1735.011 | 1735.031 | 0.0197 | 11 | 106 | 122 | LAAALPEGGVLLLENVR | 30 | 30.576 |
|  |  | 1903.966 | 1903.993 | 0.0268 | 14 | 138 | 155 | LAAVADLYVNDAFGTAHR | 22 | 0 |
|  |  | 1917.043 | 1917.059 | 0.016 | 8 | 241 | 257 | SLVEEDKLELATSLIEK |  |  |
|  |  | 1999.993 | 2000.033 | 0.0398 | 20 | 24 | 41 | ADLNVPLDDAQKITDDTR |  |  |
| 981 | Granule-bound starch synthase I | 821.3723 | 821.3282 | -0.0441 | -54 | 518 | 524 | TGFHMGR |  |  |
|  |  | 856.525 | 856.5203 | -0.0047 | -5 | 22 | 29 | SAPLSLLR |  |  |
|  |  | 1107.492 | 1107.528 | 0.036 | 33 | 336 | 344 | GCELDNIMR |  |  |
|  |  | 1350.672 | 1350.694 | 0.0218 | 16 | 550 | 561 | VVGTPAYEEMVR |  |  |
|  |  | 1368.712 | 1368.715 | 0.003 | 2 | 386 | 398 | EALQAEAGLPVDR |  |  |
|  |  | 1564.769 | 1564.776 | 0.0073 | 5 | 259 | 271 | VAFCIHNISYQGR |  |  |
|  |  | 1729.807 | 1729.82 | 0.0129 | 7 | 272 | 285 | FAFEDYPELNLSER |  |  |
|  |  | 1794.971 | 1794.924 | -0.0472 | -26 | 382 | 398 | ALNKEALQAEAGLPVDR |  |  |
|  |  | 1804.803 | 1804.835 | 0.0322 | 18 | 288 | 303 | SSFDFIDGYDTPVEGR | 44 | 96.331 |
|  |  | 1981.064 | 1981.083 | 0.0184 | 9 | 318 | 335 | VLTVSPYYAEELISGIAR |  |  |
| 1092 | Granule-bound starch synthase I | 856.525 | 856.5059 | -0.0191 | -22 | 22 | 29 | SAPLSLLR |  |  |
|  |  | 1082.537 | 1082.537 | 0.0009 | 1 | 372 | 381 | YDATTAIEAK |  |  |
|  |  | 1123.487 | 1123.475 | -0.0118 | -11 | 336 | 344 | GCELDNIMR |  |  |
|  |  | 1350.672 | 1350.704 | 0.0321 | 24 | 550 | 561 | VVGTPAYEEMVR |  |  |
|  |  | 1368.712 | 1368.73 | 0.0177 | 13 | 386 | 398 | EALQAEAGLPVDR | 21 | 0 |
|  |  | 1564.769 | 1564.815 | 0.0462 | 30 | 259 | 271 | VAFCIHNISYQGR |  |  |
|  |  | 1729.807 | 1729.863 | 0.0566 | 33 | 272 | 285 | FAFEDYPELNLSER | 29 | 11.192 |
|  |  | 1794.971 | 1795.035 | 0.064 | 36 | 382 | 398 | ALNKEALQAEAGLPVDR |  |  |
|  |  | 1804.803 | 1804.867 | 0.0646 | 36 | 288 | 303 | SSFDFIDGYDTPVEGR | 43 | 95.31 |
| 1135 | Cupin domain containing protein | 925.5465 | 925.5434 | -0.0031 | -3 | 96 | 104 | VAVLEAAPR | 26 | 0 |
|  |  | 953.4622 | 953.4628 | 0.0006 | 1 | 135 | 141 | RESFCVR |  |  |
|  |  | 1084.636 | 1084.647 | 0.0104 | 10 | 122 | 131 | EGEGVIVLLR |  |  |
|  |  | 1150.528 | 1150.539 | 0.0117 | 10 | 265 | 274 | SPYFSNNHGK |  |  |
|  |  | 1178.605 | 1178.625 | 0.0194 | 16 | 212 | 220 | EELEKVFER |  |  |
|  |  | 1223.584 | 1223.601 | 0.0175 | 14 | 301 | 311 | GSMIAPNYNTR | 20 | 0 |
|  |  | 1239.568 | 1239.591 | 0.0233 | 19 | 275 | 284 | LFELTGDECR | 36 | 72.766 |
|  |  | 1529.744 | 1529.78 | 0.0361 | 24 | 223 | 236 | EGGEITTAPEEQIR | 106 | 100 |
|  |  | 1564.776 | 1564.818 | 0.042 | 27 | 81 | 95 | FPDEQVVGAAVGGYR | 105 | 100 |
|  |  | 1813.904 | 1813.961 | 0.0573 | 32 | 221 | 236 | QREGGEITTAPEEQIR | 32 | 38.749 |
|  |  | 1884.874 | 1884.944 | 0.0706 | 37 | 49 | 65 | HGGEGGRPYHLGEESFR |  |  |
|  |  | 1890.919 | 1890.971 | 0.0522 | 28 | 245 | 264 | GGGGGSGSEWEIKPSSLTGK |  |  |
|  |  | 1986.998 | 1987.15 | 0.1519 | 76 | 223 | 240 | EGGEITTAPEEQIRELSK |  |  |
|  |  | 2028.97 | 2029.081 | 0.1106 | 55 | 105 | 121 | AFLQPSHYDADEVFYVK |  |  |
|  |  | 2218.081 | 2218.082 | 0.0012 | 1 | 142 | 163 | EGDAMVIPAGAIVYSANTHSSK |  |  |
|  |  | 2296.172 | 2296.265 | 0.0925 | 40 | 75 | 95 | FSVLERFPDEQVVGAAVGGYR |  |  |
| 1159 | Pyruvate phosphate dikinase | 864.4243 | 864.4226 | -0.0017 | -2 | 85 | 92 | SEGNKAMK |  |  |
|  |  | 970.4629 | 970.4731 | 0.0102 | 11 | 675 | 682 | SDFEGIFR | 42 | 95.019 |
|  |  | 1015.56 | 1015.546 | -0.0141 | -14 | 816 | 824 | VGTMIEIPR |  |  |
|  |  | 1072.582 | 1072.582 | -0.0005 | 0 | 683 | 692 | AMDGLPVTIR |  |  |
|  |  | 1262.656 | 1262.641 | -0.0151 | -12 | 732 | 742 | LSEVNPMLGFR |  |  |
|  |  | 1278.654 | 1278.655 | 0.0005 | 0 | 652 | 662 | QMIMASSLELR |  |  |
|  |  | 1369.584 | 1369.62 | 0.0354 | 26 | 636 | 646 | TEHMFFASDER | 23 | 0 |
|  |  | 1371.636 | 1371.631 | -0.0055 | -4 | 923 | 934 | AGLDYVSCSPFR |  |  |
|  |  | 1444.747 | 1444.757 | 0.0105 | 7 | 423 | 435 | IAVDMVNEGLVER |  |  |
|  |  | 1566.798 | 1566.79 | -0.0077 | -5 | 652 | 664 | QMIMASSLELRQK |  |  |
|  |  | 1623.805 | 1623.78 | -0.0247 | -15 | 746 | 759 | LGISYPELTEMQAR |  |  |
|  |  | 1734.796 | 1734.803 | 0.0062 | 4 | 713 | 728 | ELCSETGAAQDDVLAR | 4 | 0 |
| 1224 | 60S acidic ribosomal protein | 816.4574 | 816.4042 | -0.0532 | -65 | 96 | 102 | GDLKEVR |  |  |
|  |  | 1245.698 | 1245.696 | -0.0025 | -2 | 47 | 58 | GLRGDSIVLMGK |  |  |
|  |  | 1282.635 | 1282.625 | -0.0094 | -7 | 18 | 27 | LCQLLDEYTK | 26 | 0 |
|  |  | 1410.73 | 1410.747 | 0.0168 | 12 | 17 | 27 | KLCQLLDEYTK |  |  |
|  |  | 1411.841 | 1411.831 | -0.0093 | -7 | 153 | 165 | GTVEIITPVELIK | 48 | 99.184 |
|  |  | 1742.907 | 1742.834 | -0.0733 | -42 | 278 | 297 | FAVAAPVAADSGAAAPSAAK |  |  |
|  |  | 1939.061 | 1939.073 | 0.0122 | 6 | 28 | 45 | VLIAVADNVGSNQLQEIR | 67 | 99.99 |
|  |  | 2005.987 | 2005.97 | -0.0169 | -8 | 250 | 267 | NVLAVAVETEYSYPHADK |  |  |
|  |  | 2067.156 | 2067.157 | 0.0012 | 1 | 28 | 46 | VLIAVADNVGSNQLQEIRK |  |  |
| 1426 | Glutelin | 882.3886 | 882.3752 | -0.0134 | -15 | 44 | 49 | ECRFDR |  |  |
|  |  | 1016.523 | 1016.534 | 0.0105 | 10 | 248 | 255 | LQSQNDRR |  |  |
|  |  | 1087.528 | 1087.531 | 0.0034 | 3 | 131 | 140 | HFGLEGGSQR | 34 | 62.955 |
|  |  | 1101.642 | 1101.646 | 0.0043 | 4 | 50 | 58 | LQAFEPLRK | 30 | 9.065 |
|  |  | 1248.768 | 1248.783 | 0.0159 | 13 | 87 | 97 | IIEPQGLLLPR | 54 | 99.64 |
|  |  | 1319.678 | 1319.707 | 0.0292 | 22 | 443 | 454 | AMPVDVIANAYR |  |  |
|  |  | 1391.743 | 1391.77 | 0.0267 | 19 | 47 | 57 | FDRLQAFEPLR |  |  |
|  |  | 1404.869 | 1404.894 | 0.0252 | 18 | 86 | 97 | RIIEPQGLLLPR |  |  |
|  |  | 1471.664 | 1471.77 | 0.1065 | 72 | 298 | 309 | YNGLDENFCAIR |  |  |
|  |  | 1513.858 | 1513.895 | 0.0369 | 24 | 233 | 246 | LLSEALGVNIEVTR | 36 | 76.137 |
|  |  | 1669.96 | 1669.984 | 0.024 | 14 | 233 | 247 | LLSEALGVNIEVTRR |  |  |
|  |  | 2971.389 | 2971.568 | 0.1792 | 60 | 61 | 85 | HEAGVTEYFDEKNEQFQCTGTLVIR |  |  |
|  |  | 3015.528 | 3015.646 | 0.1172 | 39 | 267 | 290 | LIKPTITQQQEQTQDQYQQIQYHR |  |  |
| 1462 | Cupin domain containing protein | 819.4723 | 819.4648 | -0.0075 | -9 | 74 | 80 | GLFVLDR |  |  |
|  |  | 1068.543 | 1068.55 | 0.0069 | 6 | 84 | 93 | VVESEGGHVR |  |  |
|  |  | 1111.441 | 1111.453 | 0.0124 | 11 | 112 | 121 | GWSAASGCCR | 7 | 0 |
|  |  | 1133.631 | 1133.648 | 0.0163 | 14 | 74 | 83 | GLFVLDRGEK | 12 | 0 |
|  |  | 1295.674 | 1295.688 | 0.0138 | 11 | 228 | 239 | HPQSVIAGFDPK | 46 | 98.405 |
|  |  | 1470.741 | 1470.768 | 0.0268 | 18 | 158 | 169 | VGWMHKDELVEK |  |  |
|  |  | 1729.892 | 1729.929 | 0.037 | 21 | 97 | 111 | GRPWPPAAVPDPWQR |  |  |
| 1536 | Glutelin | 877.489 | 877.4889 | -0.0001 | 0 | 211 | 217 | IGQQLYR | 13 | 0 |
|  |  | 959.4694 | 959.4702 | 0.0008 | 1 | 147 | 153 | FRDEHQK |  |  |
|  |  | 1025.505 | 1025.495 | -0.0099 | -10 | 202 | 210 | DFFLAGNNK |  |  |
|  |  | 1189.538 | 1189.541 | 0.0031 | 3 | 249 | 257 | QLQCQNDQR | 0 | 0 |
|  |  | 1258.57 | 1258.565 | -0.0053 | -4 | 288 | 297 | DYGQTQYQQK | 45 | 97.654 |
|  |  | 1318.665 | 1318.681 | 0.0158 | 12 | 200 | 210 | HRDFFLAGNNK |  |  |
|  |  | 1743.856 | 1743.856 | 0.0007 | 0 | 249 | 262 | QLQCQNDQRGEIVR | 0 | 0 |
|  |  | 2895.46 | 2895.429 | -0.0305 | -11 | 263 | 287 | VEHGLSLLQPYASLQEQQQEQVQSR | |  |
| 1652 | Glutelin | 870.4427 | 870.5265 | 0.0838 | 96 | 477 | 483 | EDSRHVK |  |  |
|  |  | 947.5673 | 947.5643 | -0.003 | -3 | 108 | 115 | LVYIVQGR | 32 | 52.12 |
|  |  | 1037.476 | 1037.477 | 0.0008 | 1 | 37 | 45 | GFRGDQDSR |  |  |
|  |  | 1064.589 | 1064.593 | 0.0046 | 4 | 208 | 216 | EFFLAGKPR | 51 | 99.305 |
|  |  | 1092.514 | 1092.586 | 0.0714 | 65 | 487 | 496 | GDEMAVFAPR |  |  |
|  |  | 1220.69 | 1220.745 | 0.0552 | 45 | 207 | 216 | REFFLAGKPR |  |  |
|  |  | 1649.744 | 1649.791 | 0.047 | 28 | 64 | 77 | SEAGFTEYYNIEAR | 100 | 100 |
|  |  | 1680.845 | 1680.886 | 0.0411 | 24 | 50 | 63 | FEHLTALEATHQQR | 20 | 0 |
|  |  | 1968.931 | 1968.965 | 0.0345 | 18 | 116 | 133 | GVFGMALPGCPETFQSVR |  |  |
|  |  | 2006.967 | 2007.02 | 0.0534 | 27 | 134 | 152 | SPFEQEVATAGEAQSSIQK |  |  |
|  |  | 2105.952 | 2106.035 | 0.0823 | 39 | 217 | 233 | SSWQQQSYSYQTEQLSR | 89 | 100 |
| 1981 | Glutelin | 911.4039 | 911.3911 | -0.0128 | -14 | 208 | 214 | EQQMYGR | 15 | 0 |
|  |  | 958.47 | 958.458 | -0.012 | -13 | 247 | 254 | LQGQNDQR |  |  |
|  |  | 959.4694 | 959.4708 | 0.0014 | 1 | 143 | 149 | FRDEHQK |  |  |
|  |  | 1114.571 | 1114.566 | -0.005 | -4 | 246 | 254 | RLQGQNDQR |  |  |
|  |  | 1147.585 | 1147.599 | 0.0136 | 12 | 198 | 207 | EFLLAGNNNR |  |  |
|  |  | 1403.739 | 1403.768 | 0.0287 | 20 | 196 | 207 | QKEFLLAGNNNR | 42 | 95.029 |
|  |  | 1526.804 | 1526.834 | 0.0304 | 20 | 247 | 259 | LQGQNDQRGEIIR | 8 | 0 |

a Numbers correspond to the 2-DE gels shown in Supplemental Figure 2.
