# Supplementary figures and images for: Proteomic Analysis Reveals Proteins Involved in Seed Imbibition under Salt Stress in Rice
Source: Front Plant Sci. 2017 Jan 5;7:2006. doi: 10.3389/fpls.2016.02006 (PMC5213780; doi:10.3389/fpls.2016.02006)

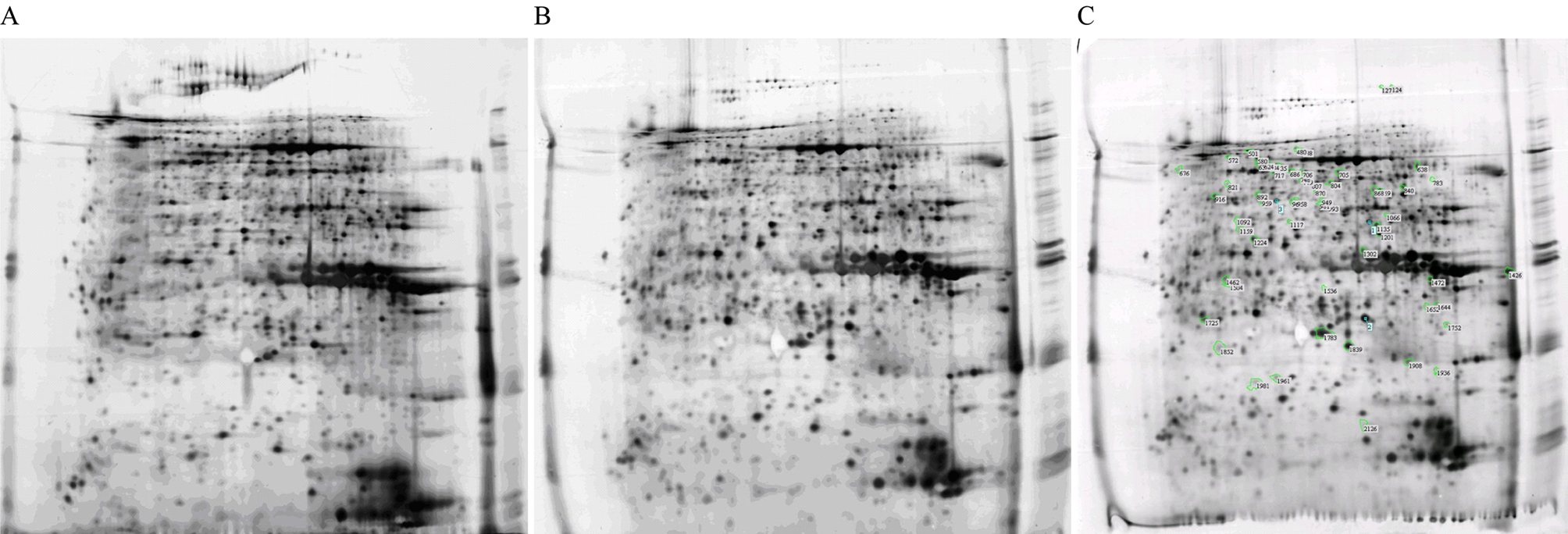

Supplement: Supplementary Figure 1 — 2-D analysis of rice proteome during seed germination under 150 mM NaCl condition. 2-D gel profiles of total proteins from dry seeds (A) and 24 h imbibed seeds (B), and the proteins statistically significant differences (P < 0.05) at least 1.5-fold between dry and 24 h imbibed seeds (C). An equal amount (100 μg) of total protein extracts was loaded in each gel. [file Image1.TIF]

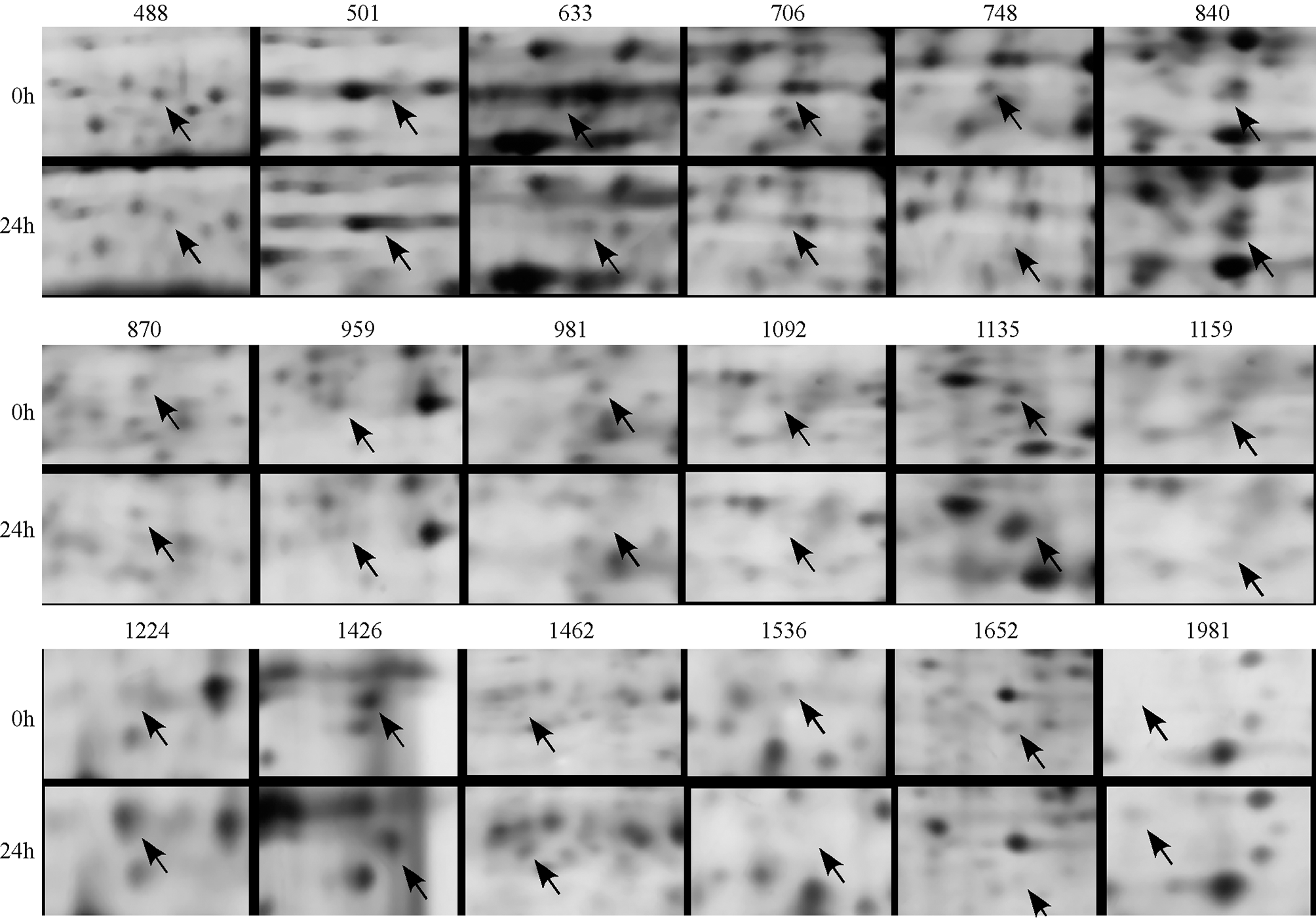

Supplement: Supplementary Figure 2 — Magnified views of the gels showing down- and up-regulated proteins statistically significant differences (P < 0.01) at least 2-fold between 24 h imbibed and dry seeds under 150 mM NaCl condition. Proteins eluted from these spots were successfully identified by MALDI-TOF-MS (see Table 1). [file Image2.TIF]
